# Vaginal metastasis of lung cancer: A case report

**DOI:** 10.1016/j.crwh.2023.e00536

**Published:** 2023-08-18

**Authors:** Federica Savasta, Michele Giana, Alessandro Libretti, Silvia Genestroni, Daniela Surico, Valentino Remorgida

**Affiliations:** a Department of Gynaecology and Obstetrics, University of Piemonte Orientale, Ospedale Maggiore della Carità, corso Mazzini 19, Novara, Italy; b Department of Oncology, University of Piemonte Orientale, Ospedale Maggiore della Carità, corso Mazzini 19, Novara, Italy

**Keywords:** Vaginal metastasis, Adenosquamous carcinoma, Lung cancer, Case report

## Abstract

Lung adenosquamous carcinoma (ASC) is a rare biphasic malignant tumor with squamous cell carcinoma (SCC) and adenocarcinoma (AC) components. ASC is reported to be aggressive; the most common metastatic sites are the regional lymph nodes and surrounding areas.

A 46-year-old woman was referred to the emergency department with a persistent dry cough. She underwent fibro-bronchoscopy and was diagnosed with an adenosquamous lung carcinoma. Other than pulmonary and lymphatic findings, a total-body computed tomography (CT) examination highlighted a hypodense formation, of about 9 mm, with a cystic appearance, at the level of the vaginal region. A biopsy performed in the posterior vaginal wall highlighted a vaginal wall flap with subepithelial localization of neoplasia, compatible with the pulmonary ASC. Oncologists took charge of the case and the patient commenced medical therapy with entrectinib. Four months later, she developed dyspnea, and high-resolution CT highlighted an increase in the pathological tissue causing bronchial occlusion. The patient underwent endobronchial stent placement and thereafter restarted therapy with entrectinib, previously stopped because of the new symptoms, and was closely monitored.

Apparently only one case of vaginal metastasis from pulmonary tumor has been previously reported, and this is the first report of vaginal metastasis from ASC. Although extremely rare, the presence of such metastasis should be considered in women with suspected vaginal neoformations.

## Highlights

• Lung adenosquamous carcinoma is a rare biphasic malignant tumor.
• lung primitive tumors may metastasize to female genital tract.
• vaginal tumors are frequently asymptomatic.
• Such metastasis should be considered if suspects of vaginal neoformations.

## Graphical abstract

**Figure:**
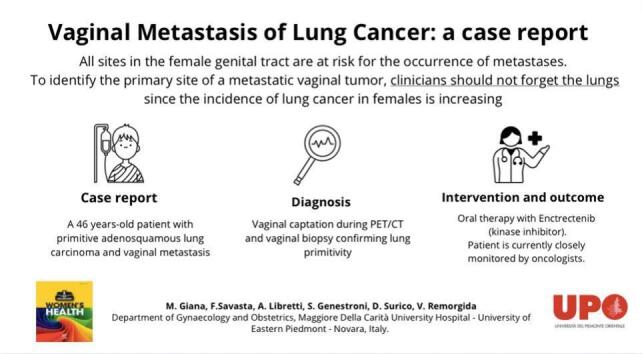

## 1 Introduction

Lung adenosquamous carcinoma (ASC) is a rare biphasic malignant tumor with squamous cell carcinoma (SCC) and adenocarcinoma (AC) components [1]. It accounts for 0.4–4% of all lung carcinomas [2,3]. It is more frequent in males, with an average age at the time of diagnosis of 68.7 years, with a higher prevalence in patients with a long history of smoking [4]. ASC is reported to be aggressive, with strong lymph nodal invasiveness and frequent metastasis at diagnosis [2]. The most common metastatic sites are the regional mediastinal lymph nodes [5], and surrounding areas, as well as liver, bones, adrenal glands, and brain [6].

There seems to be only one case of vaginal metastasis of pulmonary adenocarcinoma [7] reported in the literature and the present case appears to be the first of adenosquamous pulmonary carcinoma with vaginal metastasis reported.

## 2 Case Presentation

A 46-year-old woman was referred to the emergency department with a three-month history of persistent dry cough. She did not report fever and the white blood cell count and the c-reactive protein were within the normal range. Due to the persistence of her symptoms, a chest x-ray was performed. It showed a right basal thickening and a small effusion on the left. A chest computed tomography (CT) examination was therefore performed; it highlighted an extensive thickening of the right lower lobe and voluminous lymphadenopathies in the hilo-mediastinal stations. A PET/CT scan confirmed multiple adenopathy in the thoracic and abdominal region, together with pancreatic and bone lesions, and an irregular hyperaccumulation of ^18^F-FDG (fluorodeoxyglucose) tracer at the level of the posterior uterine wall.

The patient underwent fibro-bronchoscopy, and a biopsy revealed, at the biomolecular examination, an adenosquamous carcinoma, with co-expression of thyroid transcription factor 1 (TTF1) and p40, two markers which indicate respectively the adenocarcinoma component and the squamous component. In addition, the tumor presented proto-oncogene tyrosine-protein kinase ROS1 translocation, and programmed death-ligand 1 (PD-L1) expression.

Due to the radiologic report, she also underwent a gynaecologic examination together with a transvaginal ultrasound, which revealed a fibromatous uterus with multiple nodes and endometrial thickening. Total-body CT confirmed these findings, and also highlighted a hypodense neoformation, of about 9 mm, with a cystic appearance, at the level of the vaginal region, on the left.

An operative hysteroscopy was performed, and numerous biopsies were taken. Endometrial biopsies were negative, while the biopsy of the posterior uterine wall neoformation showed fragments of myoma. Moreover, the biopsy performed in the posterior vaginal wall highlighted a vaginal wall flap with subepithelial localization of neoplasia, compatible with pulmonary adenosquamous carcinoma as a metastasis from the patient's primary tumor.

Oncologists took charge of the case and gave indications to start medical therapy with entrectinib. Four months after having begun the medical therapy, the patient manifested a worsening of dyspnea. She therefore underwent a high-resolution CT scan (HR-CT) and suspended temporarily the medical therapy, while a pneumological evaluation was performed. The HR-CT highlighted a dimensional increase in the pathological tissue at the right hilar level, which determined marked unilateral stenosis of the lower lobar bronchus and occlusion of the bronchus for the completely atelectatic middle lobe. Mediastinal lymphadenopathies increased too, in particular a lymph node located in the upper right paratracheal area. An additional lymphadenopathy was reported at the right subpectoral level and ipsilateral supraclavicular level. An osteolytic alteration of about 1 cm appeared at the level of the sternum, interrupting the cortex on the anterior side; a disease localization had appeared at the level of the middle arch of the right V rib, of about 12 mm, and the disease localizations at the level of the fifth-sixth rib on the left increased.

The patient underwent endobronchial stent placement and was thereafter currently closely monitored, after restarting the medical therapy with entrectinib.

## 3 Discussion

Extragenital tumor metastasis of the female genital tract is rare [7]. Although various cases of pulmonary metastasis of a primitive vaginal adenocarcinoma have been reported [8,9], there has apparently been only one case described of vaginal metastasis of lung cancer [7]. In the latter, the authors reported an interesting case of a 67-year-old postmenopausal woman with a previous lung adenocarcinoma diagnosis, treated with partial lung resection. Two-years following surgery, the patient complained of increasing urinary problems, and a suspect tumor was identified with a gynecologic examination together with the imaging (ultrasound scan). Histological and immunohistochemical examinations of a vaginal excision biopsy revealed a metastatic adenocarcinoma, with the staining reactivity indicating primary lung neoplasia. The present case seems to be the first of ASC with vaginal metastasis reported.

Mazur et al. [10] analyzed the origin of metastasis in the female genital tract (Table 1). According to those authors, only 149 out of 325 cases presented extragenital primitivity. Of these, ovary and vagina were the most frequent metastatic sites for both extragenital and genital primary tumors, and the majority of the extragenital metastases were adenocarcinomas from the gastrointestinal tract. In that series, only two patients out of 149 (1.3%) presented genital tract metastasis from a primitive lung neoplasia, on the ovary and cervix. Of the twenty vaginal secondary tumors (13.4%), eleven (55%) originated from a colon/rectum primitive tumor, one (5%) from the appendix, three (15%) from breast cancer, two (10%) had an uncertain origin, and three (15%) derived from the urological tract (bladder or kidney).

**Table 1:** Female genital metastasis from extragenital tumors [10].

	Site of metastasis					
Primary site	Ovary	Salpinx	Endometrium	Cervix	Vagina	Vulva
Colon, rectum	40	1	2	2	11	0
Appendix	2	0	1	0	1	0
Stomach	6	0	1	1	0	0
Breast	46	0	2	0	3	1
Uncertain	10	0	1	0	2	1
Miscellaneous	9⁎	0	0	2⁎⁎	3⁎⁎⁎	1⁎⁎⁎⁎
Total	113	1	7	5	20	3

Total number of tumors: 149.

⁎ Jejunal adenocarcinoma (1), ileal carcinoid (1), gallbladder (1), pancreas (3), lung (1), kidney (1), rhabdomyosarcoma of parotid (1).

⁎⁎ Melanoma of thoracic skin (1), lung (1).

⁎⁎⁎ Bladder (2), kidney (1).

⁎⁎⁎⁎ Kidney (1).

Most primary vaginal tumors are squamous cell carcinomas [11], whilst other histological subtypes, such as adenocarcinomas, are extremely rare [12]. In this regard, histopathological examination is obviously essential for an accurate diagnosis. On the other hand, when the histopathological examination of a vaginal tumor shows an adenosquamous carcinoma, the possibility of a metastasis from the lungs must be considered and further analyzed.

As for clinical manifestation, vaginal metastases are frequently asymptomatic. Vaginal bleeding is the most common clinical presentation of vaginal cancer, more often postcoital or postmenopausal. Vaginal tumors may be accompanied by watery, blood‑tinged, or malodorous vaginal discharge, or a vaginal mass may be noted by the patient [13]. Other potential symptoms are related to local extension of disease, including urinary symptoms such as frequency, dysuria, hematuria, or gastrointestinal complaints such as tenesmus, constipation, or melena. Pelvic pain from extension of disease beyond the vagina is rare [14].

The treatment of carcinoma of the vagina depends primarily on histology, anatomical localization of the lesion, tumor volume, stage of the disease, and age of the patient. Different modalities of treatment can be offered to patients; however, the role of surgery is limited in primary vaginal cancer, since the primary tumor is in close proximity to the bladder, urethra, and rectum [15]. Kinase inhibitors such as entrectinib represent first-line treatment for ROS1-positive metastatic non-small cell lung cancer and NTRK gene fusion positive solid tumors. They are recommended at locally advanced stages or in metastatic tumors, such as the case presented here. Therefore, the presence of vaginal metastasis would not have implied a different approach regarding the systemic therapy, given the locally advanced stage of the primitive tumor in the mediastinal region at the time of diagnosis [16].

## 4 Conclusions

All sites in the female genital tract are at risk for the occurrence of metastasis. However, regardless of the site, the survival rate is poor [10]. Since the incidence of lung cancer in females is increasing [17], clinicians should not forget to consider vaginal metastasis. Moreover, this case confirms the importance of a multidisciplinary team, which should be always be involved in the examination of a complex case, in order to avoid missing the diagnosis.

## References

[1] Naunheim K.S., Taylor J.R., Skosey C., Hoffman P.C., Ferguson M.K., Golomb H.M. (1987). Adenosquamous lung carcinoma: clinical characteristics, treatment, and prognosis. Ann. Thorac. Surg..

[2] Li C., Lu H. (2018). Adenosquamous carcinoma of the lung. Onco Targets Ther..

[3] Rao N. (2014 Jul). Adenosquamous carcinoma. Semin. Diagn. Pathol..

[4] Maeda H., Matsumura A., Kawabata T. (2012). Adenosquamous carcinoma of the lung: surgical results as compared with squamous cell and adenocarcinoma cases. Eur. J. Cardiothorac. Surg..

[5] Li J., Zhang D.C., He J., Liu X.Y., Mu J.W., Zhang L.Z. (2009 Jul). The rule of lymph node metastasis of adenosquamous carcinoma of the lung. Zhonghua Zhong Liu Za Zhi.

[6] Kong M., Jin J., Cai X., Shen J., Ma D., Ye M., Zhu C., Freedman S., Walters K., Xu X., Chen B. (2017 Dec). Characteristics of lymph node metastasis in resected adenosquamous lung cancer. Medicine (Baltimore).

[7] Jahnke A., Domke R., Makovitzky J., Nizze H., Briese V. (2005 May-Jun). Vaginal metastasis of lung cancer: a case report. Anticancer Res..

[8] Nesme P., Bayle J.Y., Perol M., Mathevet P., Vitrey D., Guerin J.C. (1993). Pulmonary metastases from a hormone-induced clear cell adenocarcinoma of the vagina. Rev. Mal. Respir..

[9] González Casaurrán G., Simón Adiego C., Peñalver Pascual R., Moreno Mata N., Lozano Barriuso M.Á. (2011 Mar). González Aragoneses F. Surgery of female genital tract tumour lung metastases. Arch. Bronconeumol..

[10] Mazur M.T., Hsueh S., Gersell D.J. (1984 May 1). Metastases to the female genital tract. Analysis of 325 cases. Cancer..

[11] Yang J., Delara R., Magrina J., Magtibay P., Langstraat C., Dinh T., Karlin N., Vora S.A., Butler K. (2020 Nov). Management and outcomes of primary vaginal Cancer. Gynecol. Oncol..

[12] Sadatomo A., Koinuma K., Horie H., Lefor A.K., Sata N. (2015 Nov 24). An isolated vaginal metastasis from rectal cancer. Ann. Med. Surg. (Lond.).

[13] Choo Y.C., Anderson D.G. (1982 Aug). Neoplasms of the vagina following cervical carcinoma. Gynecol. Oncol..

[14] Herbst A.L., Ulfelder H., Poskanzer D.C. (1971 Apr 15). Adenocarcinoma of the vagina. Association of maternal stilbestrol therapy with tumor appearance in young women. N. Engl. J. Med..

[15] Adams T.S., Rogers L.J., Cuello M.A. (2021 Oct). Cancer of the vagina: 2021 update. Int. J. Gynaecol. Obstet..

[16] Drilon A., Siena S., Dziadziuszko R. (2020). Entrectinib in ROS1 fusion- positive non-small-cell lung cancer: integrated analysis of three phase 1-2 trials. Lancet Oncol..

[17] Domagala-Kulawik J., Trojnar A. (2020 Aug). Lung cancer in women in 21th century. J. Thorac. Dis..

